# Lung function across the life course in Kenya: a series of cross-sectional surveys

**DOI:** 10.1183/23120541.01577-2025

**Published:** 2026-07-06

**Authors:** Hellen Meme, Sophie Matu, Fred Orina, Barbara Miheso, Richard Kiplimo, Amos Ndombi, Immaculate Kathure, James Kinyanjui, Evans Amukoye, Jeremiah Chakaya, Nengjie He, Cressida Bowyer, Cindy M. Gray, Maia Lesosky, Kevin Mortimer, Sean Semple, Sarah E. West, Lindsay Zurba, Amsalu B. Binegdie, Asma El Sony, Graham Devereux

**Affiliations:** 1 Centre for Respiratory Disease Research, Kenya Medical Research Institute, Nairobi, Kenya; 2 Division of Tuberculosis and Other Lung Diseases, Ministry of Health, Nairobi, Kenya; 3 Kenya National Bureau of Statistics, Nairobi, Kenya; 4 National Heart and Lung Institute, Imperial College, London, UK; 5 Faculty of Creative and Cultural Industries, University of Portsmouth, Portsmouth, UK; 6 School of Social and Political Sciences, University of Glasgow, Glasgow, UK; 7 Cambridge Africa, Department of Pathology, University of Cambridge, Cambridge, UK; 8 Department of Paediatrics and Child Health, School of Clinical Medicine, College of Health Sciences, University of KwaZulu Natal, Durban, South Africa; 9 Respiratory Medicine, Aintree University Hospital, Liverpool, UK; 10 Institute for Social Marketing and Health, University of Stirling, Stirling, UK; 11 Stockholm Environment Institute, University of York, York, UK; 12 Education for Health Africa, Durban, South Africa; 13 Division of Pulmonary and Critical Care Medicine, Department of Internal Medicine, College of Health Sciences, Addis Ababa University, Addis Ababa, Ethiopia; 14 Epidemiological Laboratory for Public Health, Research and Development, Khartoum, Sudan; 15 Department of Clinical Sciences, Liverpool School of Tropical Medicine, Liverpool, UK

## Abstract

**Background:**

The high prevalence of COPD in sub-Saharan Africa is poorly understood. In high-income countries, COPD is the consequence of suboptimal lung growth during childhood and/or accelerated lung function decline in adult life. We have conducted cross-sectional studies to measure the lung function of children and adults in Kenya and to identify associations with age.

**Methods:**

We performed spirometry in three groups in Kenya: a random sample of schoolchildren in two districts of urban Nairobi and age/sex-stratified representative community samples of adults in Nairobi and rural Machakos. Forced expiratory volume in 1 s (FEV_1_) and forced vital capacity were expressed as z-scores using race-neutral GLI-Global reference equations.

**Results:**

The mean (95% CI) FEV_1_ z-score in Nairobi schoolchildren (n=2373, median age 10 years (IQR 8–13) 52% girls) was −0.60 (−0.64– −0.55); in Nairobi adults (n=2936, median age 32 years (24–43), 62% female) −0.49 (−0.53– −0.45); and in Machakos adults (n=1607, median age 46 years (35–59), 65% female) −0.67 (−0.72– −0.61). In adults, FEV_1_ was negatively associated with age (FEV_1_ z-score regression coefficient β −0.005/year (95% CI −0.009– −0.002) p=0.005, and there was a negative interaction between residence in Nairobi and age, β −0.006/year (95% CI −0.011– −0.001), p=0.020.

**Conclusion:**

The lung function of children and adults in Kenya was lower than predicted by race-neutral Global Lung Function Initiative (GLI)-Global reference equations. In adults, a negative association between lung function and age was greater in urban, than in rural, settings. Further work is required to identify and mitigate relevant influences.

## Shareable abstract

**Lung function was measured in Kenya using race-neutral predictive equations. The lung function of children and adults was less than predicted. In adults, a negative association between lung function and age was greater in urban, than in rural, settings.** https://bit.ly/4t4Sk13

## Background

Globally an estimated 174 million people are affected by COPD, making it the world's fourth leading cause of death and the sixth leading cause of disability [1–3]. Low- and middle-income countries (LMICs) are disproportionately burdened by COPD, with 75% of people affected by COPD and 85% of COPD deaths being in LMICs [2]. In sub-Saharan Africa (sSA) the prevalence of obstructive lung function (indicative of COPD) ranges from 1.6% to 23.1%, with a pooled prevalence of 8% (95% confidence interval (CI) 6–11%) [4–6]. Studies have also highlighted a high prevalence of restrictive lung function (usually defined as forced vital capacity (FVC) less than the lower limit of normal) in sSA, ranging from 8.4% to 72.0% [7, 8].

In high-income countries (HICs), studies into the life course of COPD have highlighted the importance of lung/airway growth in the first decades of life, peak lung function at 20–25 years and the subsequent decline due to physiological and pathological lung ageing [9]. In HICs, lung function generally tracks with age, such that infants with low lung function generally tend to have low lung function throughout the life course and are at an increased risk of COPD in adult life [9–11]. Whether these life course models of lung function are applicable to sSA and the high burden of obstructive and restrictive lung function is an unanswered question because of minimal local data.

In response to the prioritisation of COPD and asthma by the Kenyan Government [12], our team has been able to conduct cross-sectional surveys of lung function in Kenyan schoolchildren, community adults and respiratory patients, using the same staff, training and spirometry quality assurance [13, 14]. The study of 174 patients attending a hospital clinic with chronic respiratory symptoms, in whom tuberculosis had been excluded, showed that the most frequent abnormal lung function pattern was obstructive (24.7%) with the restrictive pattern being less frequent (15.5%) [14]. The aims of the study presented here were firstly, to measure the lung function of children and adults in Kenya and to compare this with race-neutral Global Lung Function Initiative (GLI-Global) reference equations [15, 16] and secondly, because of our interest in the “life course” of airways disease, we wished to identify associations between lung function and age and whether these differed between rural/urban settings. To facilitate comparisons with published studies we also present race- and ethnicity-based z-scores using GLI-2012 reference equations [17].

## Materials and methods

### Study setting

Kenya (population 56 million) is a lower middle-income country with a per capita income of 1952 USD per year [18]. Between 2018 and 2021, in collaboration with international partners, the Centre for Respiratory Disease Research, Kenya Medical Research Institute (KEMRI) led two cross-sectional studies in Kenya that measured lung function:

1) “Schoolchildren study”: The Tupumue study compared schoolchildren in two socioeconomically contrasting but geographically close districts of Nairobi [13].
2) “Community adult study”: Compared adults in urban Nairobi and rural Machakos.

Schoolchildren were recruited between January 2020 and November 2021, with COVID-19 suspending recruitment between March 2020 and April 2021. Community adults were recruited between January 2018 and June 2021, with recruitment suspended by COVID-19 between March 2020 and January 2021.

### Community involvement, sampling and recruitment

Recruitment to the schoolchildren study has been published [13]. Briefly, after community sensitisation using novel arts-based methodologies, children (≤18 years) attending schools in the Nairobi communities of Mukuru (an informal settlement) and Buruburu (a more affluent suburb) were randomly selected through multi-stage cluster sampling (schools, then classes, with all children from selected classes included) from a sampling frame of all schools.

The community adult study was a cross-sectional study of adults in Nairobi and Machakos. Nairobi is densely populated (≈4 million residents) with ambient air pollution exceeding World Health Organization (WHO) recommended air quality guidelines because of high traffic volumes and industrial emissions [19]. Machakos County (population ≈1.4 million) lies 60 km southeast of Nairobi; it is predominantly rural with most people reliant on agriculture. Ambient pollution is lower in Machakos than Nairobi; however, household air pollution is likely to be greater because of widespread biomass fuel use for cooking [19, 20]. The sampling strategy is described in detail in the supplementary methods. In brief, Kenya's National Bureau of Statistics (KNBS) National Sample Survey & Evaluation Programme (NASSEP-V) database was used to randomly identify age and sex representative samples of all residents in Nairobi and Machakos Counties [21]. In total, 5112 households were randomly selected in Nairobi and 1548 households were randomly selected in Machakos. Local administrative leaders (chiefs, sub-chiefs) and village elders were briefed about the study, who then informed their communities through routine channels of communication.

### Questionnaires

In the schoolchildren study, field workers administered questionnaires to parents/guardians of children aged ≤12 years and to children aged ≥13 years. The Global Asthma Network-based questionnaire included demographics, household asset-based wealth score, respiratory symptoms and asthma diagnosis/treatment [22, 23]. For the community adult study, fieldworkers administered a questionnaire based on the Burden of Obstructive Lung Disease (BOLD) core questionnaire [24].

### Spirometry

The Easy On-PC Spirometer (NDD Medizintechnik AG, Switzerland) with on-screen incentive software was used for the schoolchildren study. For the community adult study, the EasyOne Spirometer (NDD Medizintechnik AG) was used. Technicians trained by Education for Health Africa and certified by the Pan African Thoracic Society conducted spirometry following American Thoracic Society/European Respiratory Society recommendations [25, 26]. Up to eight forced exhalations were performed whilst sitting and wearing nose clips. Internal and external assessors reviewed all spirometry traces. Measurements graded A–C for acceptability and repeatability were selected for analysis [25]. For the community adult study spirometry was measured before and after 400 µg salbutamol was administered *via* metered dose inhaler and spacer.

### Statistical considerations

Two self/parentally-reported parameters of asthma prevalence were measured: “wheeze in the last 12 months” and “doctor diagnosed asthma” [27]. COPD was quantified by lung function and self-report of “doctor diagnosis of COPD”. Spirometry parameters were forced expiratory volume in 1 s (FEV_1_), FVC and FEV_1_/FVC ratio expressed as z-scores using race-neutral Global Lung Function Initiative (GLI-Global) reference equations [15, 16] and GLI-2012 reference equations for African-American ethnicity [17]. Participants’ lung function was categorised: normal (post-bronchodilator FEV_1_ ≥ lower limit of normal (LLN), FVC ≥LLN, FEV_1_/FVC ≥LLN); obstructive only (post-bronchodilator FEV_1_/FVC <LLN, FVC ≥LLN); restrictive only (FVC <LLN, FEV_1_/FVC ≥LLN) and mixed (both obstructive and restrictive). For schoolchildren pre-bronchodilator values were used and the data from the two communities combined because there were no significant lung function differences between them [13]. For all studies, the prevalence of spirometry patterns (normal, obstructive only, restrictive only, and mixed) were age- and sex-adjusted to give estimates in line with local age/sex distributions reported in the 2019 Kenyan Census [28]. For adults, body mass index (BMI) was calculated in the usual way and for children BMI centiles were calculated [29]. Statistical tests for comparisons between groups used Fisher's exact-test/Chi-square or t-tests/Wilcoxon rank-sum as appropriate.

Separate linear regression models were fit to lung function outcomes for schoolchildren and community adult studies, using individual age as the independent variable. Study setting (urban/rural) was fit as an interaction term with age, including the main effects, in the community adult study only, in order to estimate possible effect modification by urban/rural setting. Estimated coefficients and 95% confidennce intervals were used to generate figures of average linear trend. These terms were included as the minimum set of variables required to accurately estimate the age association with lung function. Statistical analysis was carried out in R v4.5.0 (www.R-project.org/). Sample size considerations for the schoolchildren study are presented elsewhere [13]. Briefly, with spirometry data from 800 children in each community the study would have 80% power, with α=0.05, to detect z-score differences of ±0.14 between the communities. For the community adult study in Nairobi, a sample size of 2640 gave 10% precision to estimate COPD prevalence (estimated at 15%) with 20% nonresponse. In Machakos a sample size of 1924 gave 10% precision to estimate COPD prevalence (estimated at 10.5%) with 20% nonresponse.

### Ethics

All studies were approved by the KEMRI Scientific and Ethics Review Unit. The Liverpool School of Tropical Medicine Research Ethics Committee also approved the schoolchildren study. For children (<18 years) parents/guardians provided written consent and children provided written assent. Adults (≥18 years) provided written informed consent.

## Results

The number of participants involved in the studies and their demographic details are presented in table 1 and supplementary figure S1. In total, 2373 (69% of 3450 invited) children aged 4–18 years attending nine primary and three secondary schools took part. In Nairobi 2936 (41%) of the 7169 adults sampled participated, and in Machakos 1607 (68%) of the 2355 adults sampled took part. The reasons for nonparticipation in Nairobi were: not being present (3811; 90%); too young (344; 8%); or declining to take part (78; 2%). In Machakos 99% (737) of the 748 not participating were not present in the house, 8 (1%) were too young and 3 (0.4%) declined taking part. Analysis of the NASSEP-V database indicated that adult participants were more likely to be female and older than nonparticipants: in Nairobi 62% of participants and 46% of nonparticipants were female (p<0.001), in Machakos corresponding figures were 65% and 44% (p<0.001). The mean±sd age of participants in Nairobi was 34.8±13.0 years and nonparticipants 20.5±15.3 years (p<0.001); in Machakos the corresponding figures were 47.2±16.4 years and 28.5±21.7 years (p<0.001). Current tobacco smoking in Machakos (10.2%) was more frequent than in Nairobi (5.6%), although pack-year consumption of current smokers was low: 8.4 (95% CI 7.0–9.8) *versus* 5.0 (4.1–5.9), p<0.001. The mean BMI centile of the schoolchildren was 25.3 (25.0–25.6) with none being under- or overweight using WHO cut-offs [29]. The BMI profiles of the adults is outlined in table 1.

**TABLE 1:** Demographic details, respiratory symptoms and diagnoses of participants in the cross-sectional studies in Kenya

	Tupumue schoolchildren study – Nairobi	Community adult study	
		Nairobi	Machakos
**Participants, n**	2373	2936	1607
**Female, n (%)**	1240 (52.3%)	1820 (62.0%)	1048 (65.2%)
**Age years, median (IQR), (min, max)**	10 (8–13), (4, 18)	32 (24–43), (18, 88)	46 (35–59), (18, 98)
**Household asset score, median (IQR)^#^**	3 (3–6)	4 (3–5)	2 (1–3)
**Body mass index kg·m^−2^**			
Mean (95% CI)		25.6 (25.4–25.8)	24.7 (24.5–25.0)
Underweight (<18.5), n (%)		158 (5.6%)	189 (12.0%)
Normal (18.5–24.9), n (%)		1363 (47.9%)	704 (44.7%)
Overweight (25.0–29.9), n (%)		730 (25.7%)	399 (25.3%)
Obese (30.0–39.9), n (%)		553 (19.4%)	263 (16.7%)
Severely obese (≥40), n (%)		41 (1.4%)	20 (1.3%)
**Inhaled exposures**			
Current smoker, n (%)	8 (0.3%)	165 (5.6%)	164 (10.2%)
Tobacco pack-years, median (IQR)		0 (0–0)	0 (0–0)
Tobacco pack-years, mean (95% CI)		0.44 (0.38–0.53)	1.23 (1.02–1.44)
Tobacco smoker in the home, n (%)	249 (10.5%)	44 (1.5%)	37 (2.3%)
Solid fuel for cooking, n (%)	155 (6.5%)	145 (10.2%)	107 (19.1%)
**Symptoms, n (%)**			
Wheeze in last 12 months^¶^	190 (8.1%)	376 (12.8%)	156 (9.7%)
SOB hurrying/slight incline^+^		231 (7.9%)	131 (8.2%)
SOB dressing, unable to leave house^§^		3 (0.1%)	4 (0.2%)
Chronic bronchitis symptoms^ƒ^		44 (1.5%)	47 (2.9%)
**Diagnoses, n (%)^##^**			
Doctor diagnosed asthma	46 (1.9%)	160 (5.4%)	80 (5.0%)
Doctor diagnosed emphysema		5 (0.2%)	2 (0.1%
Doctor diagnosed chronic bronchitis		11 (0.4%)	12 (0.7%)
Doctor diagnosed COPD		5 (0.2%)	4 (0.2%)
Ever diagnosed with TB	13 (0.5%)	126 (4.3%)	64 (4.0%)

Empty cells indicate that these data were not collected for this study. SOB: short of breath; TB: tuberculosis; DK: don't know. ^#^: household asset score as described by Townend *et al.* [23], range 0 (none of listed assets) to 10 (all 10 listed assets); ^¶^: has this child/you had wheezing or whistling in the chest in the past 12 months? (Y/N/DK); ^+^: are you troubled by shortness of breath when hurrying on the level or walking up a slight hill? (Y/N/DK); ^§^: are you too short of breath to leave the house or short of breath on dressing or undressing? (Y/N/DK); ^ƒ^: derived from “Do you bring up this phlegm on most days for as much as three months each year?” (Y) and “For how many years have you had this phlegm?” (≥2 years); ^##^: has a doctor ever told you that you/child have…(Y/N/DK).

### Symptoms, diagnoses, treatment

Table 1 presents the symptoms and diagnoses of the study participants. “Wheezing in the previous 12 months” (recent wheeze) was reported more frequently by adults than schoolchildren, and Nairobi adults reported more “recent wheeze” than Machakos adults. Machakos adults reported more dyspnoea than Nairobi adults. Asthma was the most common respiratory diagnosis (1.9% schoolchildren, 5.3% adults). Diagnosed COPD was reported rarely (0.2% adults).

### Spirometry

**FIGURE 1:**
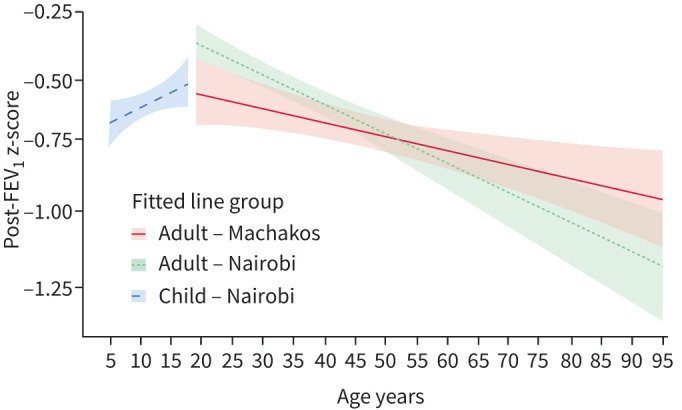
Regression lines (95% confidence intervals) for GLI-Global forced expiratory volume in 1 s (FEV_1_) z-scores in children and adult studies in Nairobi and Machakos.

**FIGURE 2:**
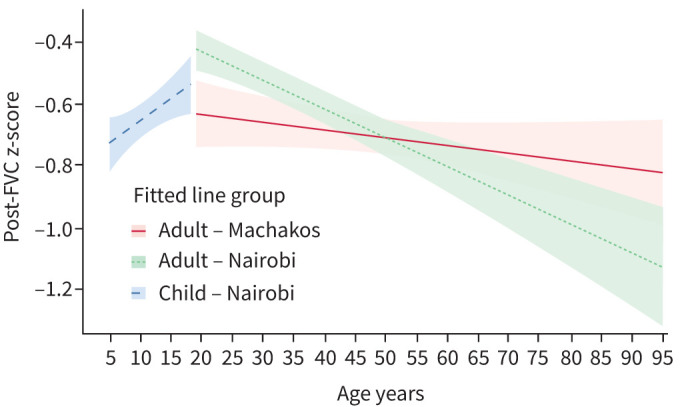
Regression lines (95% confidence intervals) for GLI-Global forced vital capacity (FVC) z-scores in children and adult studies in Nairobi and Machakos.

The spirometry aspects of the studies are presented in tables 2 and 3, supplementary tables S1–S3, figures 1 and 2 and supplementary figures S2 and S3.

**TABLE 2:** Spirometric characteristics of participants in the cross-sectional studies in Kenya GLI-Global and GLI-2012

	Schoolchildren study – Nairobi	Community adult study	
		Nairobi	Machakos
**Participants, n**	2373	2936	1607
**Spirometry**			
Valid spirometry, n (%)	1622 (68.4%)	2377 (80.9%)	1342 (83.5%)
FEV_1_ L, mean (95% CI)	2.01 (1.97–2.04)	2.90 (2.88–2.93)	2.46 (2.42–2.50)
FVC L, mean (95% CI)	2.26 (2.23–2.30)	3.40 (3.37–3.43)	2.98 (2.94–3.02)
FEV_1_/FVC, mean (95% CI)	0.888 (0.886–0.891)	0.852 (0.849–0.854)	0.822 (0.818–0.826)
**Spirometry GLI-Global**			
FEV_1_ z-score, mean (95% CI)	−0.595 (−0.640– −0.551)	−0.489 (−0.533– −0.446)	−0.665 (−0.720– −0.610)
FVC z-score, mean (95% CI)	−0.642 (−0.685– −0.598)	−0.548 (−0.590– −507)	−0.687 (−0.739– −0.635)
FEV_1_/FVC z-score, mean (95% CI)	0.110 (0.065–0.156)	0.159 (0.122–0.195)	0.051 (0.001–0.101)
**Spirometry pattern GLI-Global, n (%)^#^**			
Normal	1363 (84.4%)^#^	2021 (86.1%)^#^	1089 (82.6%)^#^
Obstructive only^¶^	37 (2.56%)^#^	47 (1.76%)^#^	38 (2.10%)^#^
Restrictive only^+^	173 (10.2%)^#^	243 (9.50%)^#^	161 (9.46%)^#^
Mixed obstruction/restrictive	2 (0.11%)^#^	16 (0.60%)^#^	24 (1.3%)^#^
**Spirometry GLI-2012**			
FEV_1_ z-score, mean (95% CI)	0.316 (0.248–0.373)	0.106 (0.058–0.154)	−0.110 (−0.172– −0.049
FVC z-score, mean (95% CI)	0.284 (0.234–0.334)	−0.018 (−0.063–0.027)	−0.189 (−0.246– −0.132)
FEV_1_/FVC z-score, mean (95% CI)	0.043 (−0.003–0.088)	0.191 (0.155–0.227)	0.092 (0.041–0.142)
**Spirometry pattern GLI-2012, n (%)^#^**			
Normal	1521 (93.1%)^#^	2163 (91.7%)^#^	1179 (89.1%)^#^
Obstructive only^¶^	51 (3.55%)^#^	47 (1.82%)^#^	44 (2.29%)^#^
Restrictive only^+^	39 (2.67%)^#^	132 (5.81%)^#^	86 (6.49%)^#^
Mixed obstructive/restrictive	0	13 (0.43%)^#^	17 (0.95%)^#^

GLI: Global Lung Function Initiative; FEV_1_: forced expiratory volume in 1 s; FVC: forced vital capacity. ^#^: percentages age- and sex-adjusted calculated using local data from the 2019 Kenya census; ^¶^: FEV_1_/FVC <LLN and FVC ≥LLN; ^+^: FVC <LLN and FEV_1_/FVC ≥LLN.

**TABLE 3:** Results of linear regression analysis relating FEV_1_ and FVC z-scores to age and study location GLI-Global

	FEV_1_ z-score			FVC z-score		
Population/Variable	Regression coefficient β	95% CI	p-value	Regression coefficient β	95% CI	p-value
**GLI-Global**						
Nairobi children (Tupumue) n=1622						
Age/year	0.012	−0.001–0.025	0.060	0.015	0.017–0.025	0.017
Adults (Nairobi *versus* Machakos) n=3719						
Age/year	−0.005	−0.009– −0.002	0.005	−0.002	−0.006–0.001	0.160
Nairobi *versus* Machakos	0.309	0.084–0.534	0.007	0.333	0.123–0.543	0.019
Nairobi×age interaction	−0.006	−0.011– −0.001	0.020	−0.007	−0.011– −0.002	0.005
**GLI-2012**						
Nairobi children (Tupumue) n=1622						
Age/year	−0.039	−0.054– −0.025	<0.001	−0.046	−0.061– −0.032	<0.001
Adults (Nairobi *versus* Machakos) n=3719						
Age/year	−0.005	−0.009– −0.001	0.012	−0.001	−0.004–0.003	0.696
Nairobi *versus* Machakos	0.411	0.162–0.660	0.001	0.454	0.226–0.683	<0.001
Nairobi×age interaction	−0.008	−0.013– −0.002	0.006	−0.009	−0.014– −0.004	0.001

FEV_1_: forced expiratory volume in 1 s; FVC: forced vital capacity; GLI: Global Lung Function Initiative.

#### Schoolchildren

In total, 1655 of the 2373 schoolchildren attempted spirometry, of which 1622 were acceptable/reproducible; the main reason for not attempting spirometry was that many children for whom questionnaire data were obtained from parents before the COVID-19 pandemic could not be identified post-COVID-19 (supplementary figure S1). As detailed elsewhere and in supplementary table S1, although children unable/unwilling to provide acceptable spirometry were younger, they were otherwise similar demographically and symptomatically to those providing acceptable spirometry [13].

Using race-neutral GLI-Global, the mean (95% CI) FEV_1_ and FVC z-scores for the Kenyan schoolchildren were −0.595 (−0.640– −0.551) and −0.642 (−0.685– −0.598), respectively; there was no sex difference (supplementary table S2). FEV_1_ and FVC z-scores were substantially higher by ≈0.9 z-score units using race and ethnicity-based GLI-2012 (table 2). Using GLI-2012, 93.1% of the schoolchildren had normal spirometry; however, with GLI-Global 84.4% had normal spirometry, with most of the increase in abnormal spirometry being restrictive (10.3%); there was a small (1%) reduction in obstructive pattern (to 2.56%) with GLI-Global.

#### Community adults

In total, 4448 (97.9%) of 4543 participants attempted spirometry, with 3719 (81.9%) producing acceptable and reproducible spirometry; supplementary figure S1 provides more details. Although adults providing acceptable spirometry were proportionately more likely to be from Machakos (p=0.033), they were very similar demographically and symptomatically to those unable/unwilling to provide acceptable spirometry (supplementary tables S1 and S3).

Using race-neutral GLI-Global, the mean (95% CI) FEV_1_ z-scores for adults living in Nairobi and Machakos were −0.489 (−0.533– −0.446) and −0.665 (−0.720– −0.610) respectively; a similar pattern was evident for FVC (table 2). FEV1 z-scores were substantially higher (≈0.5 z-score units) using race- and ethnicity-based GLI-2012 (table 2). Using GLI-2012, 91.7% of Nairobi adults and 89.1% of Machakos adults had normal spirometry; however, when GLI-Global was used, 86.1% of Nairobi adults and 82.6% of Machakos adults had normal spirometry, with most of the increase in abnormal spirometry being restrictive, the obstructive pattern remaining essentially unchanged. On average, Nairobi community adults had higher mean (95% CI) FEV_1_ (by 0.309 (0.084–0.534)) and FVC z-scores (by 0.333 (0.123–0.543)) than Machakos community adults when using GLI-Global reference equations (table 3); these differences were somewhat greater using race- and ethnicity-based GLI-2012 African-American reference equations (table 3). The prevalence of obstructive, restrictive and mixed obstructive/restrictive spirometric patterns in community adults were more common in Machakos than Nairobi. Although women had lower FEV_1_ z-scores in Nairobi on univariate analysis (supplementary table S2), there was no independent association with sex after adjustment for age.

### Lung function and age

Race-neutral GLI-Global FVC (but not FEV_1_) z-scores of Nairobi schoolchildren were positively associated with age (figures 1 and 2, table 3). In contrast, their race and ethnicity-based GLI-2012 FEV_1_ and FVC z-scores were not only significantly higher but were also negatively associated with age (table 3, supplementary figures S2 and S3).

In adults, race neutral GLI-Global derived FEV_1_ z-scores were significantly higher in Nairobi than in Machakos, and FEV_1_ z-scores were negatively associated with age; however, the negative association between FEV_1_ z-scores and age was significantly greater in Nairobi than in Machakos with a significant negative interaction with age in Nairobi (figures 1 and 2, table 3). In adults, race-neutral GLI-Global derived FVC z-scores were significantly higher in Nairobi than in Machakos, and there was a significant negative association between FVC z-scores with age in Nairobi but not in Machakos (figures 1 and 2, table 3). The mean (95% CI) age of adults with spirometry consistent with COPD in Nairobi was 40.2 years (36.2–44.1) and in Machakos 57.0 years (53.0–61.1) (p<0.001), and for restrictive spirometry 38.1 years (36.5–39.7) in Nairobi and 48.4 years (46.0–50.8) in Machakos (p<0.001).

Use of GLI-2012 for the community adult studies significantly lowered the FEV_1_ and FVC z-scores and increased the magnitude of the difference between Nairobi and Machakos; however, the associations with age did not appreciably differ (table 3, supplementary figures S2 and S3).

## Discussion

We have conducted cross-sectional surveys measuring spirometry in children and adults in Kenya. In Nairobi, the mean FEV_1_ and FVC z-scores of children were significantly lower than race-neutral GLI-Global predicted values and FVC z-scores were positively associated with age; there were no associations with sex. In adults, the mean FEV_1_ and FVC z-scores of adults in urban Nairobi and rural Machakos were significantly lower than race-neutral GLI-Global predicted values with mean FEV_1_ and FVC z-scores being higher in Nairobi than in Machakos. There were negative associations between z-scores and age that differed between Nairobi and Machakos, such that the difference between urban and rural adult lung function differed with age: FEV_1_ and FVC z-scores were significantly greater in Nairobi below ≈55 years, but above 55 years, FEV_1_ and FVC z-scores were generally higher in Machakos. We also noted that when FEV_1_ and FVC z-scores were derived using race- and ethnicity-based GLI-2012 predictive equations, mean FEV_1_ and FVC z-scores for Nairobi children and adults were significantly greater than predicted, suggesting that the lung function of children and adults in Nairobi was significantly better than the lung function of the GLI-2012 African-American reference population. Superficially this seems somewhat implausible given the substantial exposure to adverse socioeconomic, early life and environment risk factors for poor respiratory health in Kenya; however, these GLI-2012-based findings are consistent with the much lower prevalence of wheezing symptoms in the Kenyan schoolchildren compared to African-American children [30]. It was also noted that GLI-Global increased the prevalence of abnormal and restrictive spirometry when compared with GLI-2012, with the prevalence of obstructive spirometry remaining essentially unchanged or slightly reduced.

When compared with sSA studies measuring lung function in general populations of children with GLI-2012 as reference, the mean (GLI-2012 derived) FEV_1_ and FVC z-scores in Nairobi school children were higher, and obstructive and restrictive lung function deficits less frequent than in studies of children in other sSA countries [31–36]. Relatively few studies have assessed lung function in general populations aged ≥18 years in Africa using GLI (usually GLI-2012) as reference. When compared with studies in urban Cameroon and rural Malawi, obstructive and restrictive patterns were less frequent in our Kenyan adults [7, 37–39].

In Nairobi, our previous work has shown that obstructive lung function predominates in respiratory patients [14]. It is generally understood that the risk of obstructive spirometry/COPD is increased by failure to attain optimal maximal lung function in early adulthood and/or there is accelerated lung function decline in adult life [9–11]. Although not a cohort study, our cross-sectional findings of reduced childhood lung function suggest that children in Nairobi could be at increased risk of developing COPD because of failure to achieve maximal lung growth. Childhood factors that adversely affect lung function include prematurity, low birthweight, early wheezing, asthma, allergic sensitisation, allergic rhinitis, lower respiratory tract infections, family history of asthma, maternal smoking during pregnancy, air pollution, second-hand smoke exposure and low body weight, some of which are particularly pertinent in the informal settlement from which half of the study children were recruited. We also noted that symptoms of asthma (recent wheeze) and spirometric evidence of COPD greatly exceeded the diagnosis of asthma and COPD. This most likely reflects the lack of readily available and accessible lung function testing for COPD, issues of stigma associated with an asthma diagnosis in Kenya, and the lack of available and affordable treatment for asthma and COPD [40, 41].

In our community adult study, the negative association between FEV_1_ and FVC z-scores and age was significantly greater in urban Nairobi than in rural Machakos. Consequently, although adults in Machakos typically commence adult life with reduced FEV_1_ and FVC (compared to Nairobi), the association between FEV_1_ and FVC and age in Nairobi is such that after the age of about 55 years, FEV_1_ and FVC z-scores are higher in Machakos. When compared with Machakos, it appears that adults in Nairobi are at increased risk of developing COPD because FEV_1_ and FVC decline more rapidly with age, possibly as a result of higher levels of ambient air pollution [19], occupational exposures, dietary factors and a more sedentary lifestyle. Paradoxically, in Machakos, overall FEV_1_ and FVC z-scores were lower and the age-, sex-adjusted prevalences of obstructive and restrictive patterns were more frequent than in Nairobi. This most likely reflects the older age profile of adults in Machakos compared with Nairobi, and it is noticeable that adults with obstructive spirometry in Nairobi were on average 17 years younger than in Machakos [28]. If confirmed by longitudinal studies, the negative association between FEV_1_ and age in Nairobi is likely to have clinical consequences because COPD related to rapid FEV_1_ decline has been associated with an increased risk of respiratory and all-cause mortality when compared with COPD developing through low FEV_1_ in early adult life [42].

Our study has several strengths. First, the children and adult studies had a large population-based design with spirometry patterns being age- and sex-adjusted to 2019 Kenya census data. Secondly, a single team conducted spirometry to international standards with lung function values referenced against race-neutral GLI-Global equations as recommended [15, 16]. Thirdly, LLNs were used to identify obstructive and restrictive patterns, helping to prevent overdiagnosis of obstructive and restrictive patterns [43]. The main study limitation is the cross-sectional study design and the likelihood of “cohort effects” with successive birth cohorts being born with smaller or larger lungs than preceding birth cohorts. Longitudinal cohort studies spanning the life course are needed in Africa to identify lung function trajectories and to identify important influences. Secondly, we collected limited exposure data and minimal/no data on early life exposures and other risk factors such as preterm birth, childhood infections, admissions for respiratory disease and other relevant chronic illness. Thirdly, we could not measure post-bronchodilator spirometry in schoolchildren as this was locally unacceptable; instead we used pre-bronchodilator spirometry in schoolchildren which may overestimate obstruction. Fourthly, the study populations of the schoolchildren and adults in Nairobi were somewhat different socioeconomically, with 53.8% of the study sample attending schools in the informal settlement of Mukuru. Fifthly, participation in the community adult study was somewhat biased with those aged 20–39 years overrepresented in Nairobi and underrepresented in Machakos, possibly the consequence of younger people migrating to urban areas and older urban citizens retiring to rural areas. This selection bias may have influenced prevalence estimates; however, we calculated age- and sex-adjusted prevalences to mitigate this.

### Conclusions

In this series of cross-sectional surveys in Kenya, the lung function of children and adults in urban Nairobi and rural Machakos (expressed as race-neutral GLI-Global z-scores) was lower than predicted. The use of race-neutral GLI-Global resulted in notably different findings when compared with the use of race- and ethnicity-based GLI-2012 reference equations. The current model of the life course of COPD developed in HICs appears to have relevance in the Kenyan context, with children having lower than predicted lung function, and in adults there are differences in the negative association between FEV_1_ and age. However, local longitudinal studies are needed to confirm these cross-sectional data and to identify and mitigate relevant early life, socioeconomic and environmental influences.

## Data Availability

Individual de-identified participant data that underlie the results reported in this article, along with data dictionary and study protocols, are available upon reasonable request. Please contact the corresponding author.

## Supplementary material

**Please note:** supplementary material is not edited by the Editorial Office, and is uploaded as it has been supplied by the author. Supplementary material 01577-2025.SUPPLEMENT
