# “Lung function across the life course in Kenya: a series of cross-sectional surveys.” Hellen Meme, Sophie Matu, Fred Orina, Barbara Miheso, Richard Kiplimo, Amos Ndombi, Immaculate Kathure, James Kinyanjui, Evans Amukoye, Jeremiah Chakaya, Nengjie He, Cressida Bowyer, Cindy M. Gray, Maia Lesosky, Kevin Mortimer, Sean Semple, Sarah E. West, Lindsay Zurba, Amsalu B. Binegdie, Asma El Sony and Graham Devereux. *ERJ Open Res* 2026; 12: 01577-2025.

**DOI:** 10.1183/23120541.51577-2025

**Published:** 2026-08-03

This article was originally published with an error in the author list. Jeremiah Chakaya was inadvertently changed to “Jeragingh Chakaya” during copy-editing. The correct author list is shown above. This has also been corrected in the article itself. We apologise for this error.

