# Supplementary material for: Lung function across the life course in Kenya: a series of cross-sectional surveys
Source: ERJ Open Res. 2026 Jul 6;12(4):01577-2025. doi: 10.1183/23120541.01577-2025 (PMC13334340; doi:10.1183/23120541.01577-2025)

SUPPLEMENTAL MATERIALS

Lung function across the life course in Kenya: a series of cross-sectional surveys.

Hellen Meme, Sophie Matu, Fred Orina, Barbara Miheso, Richard Kiplimo, Amos Ndombi, Immaculate Kathure, James Kinyanjui, Evans Amukoye, Jeremiah Chakaya, Nengjie He, Cressida Bowyer, Cindy M Gray, Maia Lesosky, Kevin Mortimer, Sean Semple, Sarah E West, Lindsay Zurba, Amsalu B Binegdie, Asma El Sony, Graham Devereux

CONTENTS

Supplemental methods: Community adult studies sampling strategy Page 3

Figure S1: Diagram illustrating participants involvement in the studies of schoolchildren and community adults. Page 5

Table S1: Characteristics of those with and without acceptable/reproducible spirometry individual studies. Page 6

Table S2: GLI-global derived FEV_1 and FVC z-scores for males and females for the cross-sectional studies Page 7

Table S3: Characteristics of those with and without acceptable/reproducible spirometry combined community adult studies Page 8

Figure S2: Regression lines (95% CI) for GLI-2012 FEV_1 z-scores in children and adult studies in Nairobi and Machakos Page 9

Figure S3: Regression lines (95% CI) for GLI-2012 FVC z-scores in children and adult studies in Nairobi and Machakos Page 10

SUPPLEMENTAL METHODS

Community adult studies sampling strategy

Age and sex representative samples of all residents living in Nairobi and Machakos Counties were obtained using a stratified cluster sampling strategy.

The National Sample Survey & Evaluation Programme (NASSEP V) is the sampling frame the Kenya National Bureau of Statistics (KNBS) operates to conduct household surveys throughout the country. NASSEP V was used to determine the number of clusters, and number of households (HH) per cluster to attain the desired sample size of 2,640 individuals in Nairobi County which was divided as follows; 660 individuals in Nairobi for each of the following categories: men 18-39 years, women 18-39 years, men ≥40 years and women ≥40 years.

To attain a sample of 2,640, initially 4,800 households were randomly selected comprising 150 clusters with 32 households per cluster. For men 18-39 years - 947 HH (every fourth household) would be approached, for women 18-39 years - 893 HH (every fifth household), for men ≥40 years - 2,700 HH (every second household) and women ≥40 years - 4,500 HH.

Based on these initial calculations, a pilot study was conducted in four randomly selected clusters representing diverse settings within Nairobi County. Thirty two households were selected per cluster with recruitment of individual categories randomly assigned as follows: men 18-39 years - 8 HH, women 18-39 years - 18 HH, men ≥40 years - 20 HH, women ≥40 years - 32 HH. The results from the pilot study necessitated adjustment to the number of randomly selected clusters (to 142), as well as the households per cluster (36 HH), resulting in a final sample size of 5,112 households. The distribution across age groups was: 2,556 HHs for 18-39 year olds and 5,112 HH for ≥40 year olds.

For Machakos County, a similar process was undertaken to achieve the sample size of 1,924 comprising 549 men 18-39 years, 584 women 18-39 years, 368 men ≥40 years and 423 women ≥40 years. In total 43 clusters with 36 HH per cluster were randomly selected, with all 1,548 selected households being approached.

Mapping procedures

Permission was sought and obtained from the County Executive Committee (CEC) Health to conduct research within Nairobi and Machakos counties. Following this, the research team visited the KNBS County Statistics Officer (CSO) to brief them on the study procedures and request the assignment of a KNBS enumerator to assist during mapping. A courtesy call was then made to the National Government Administration Office (NGAO) in the company of the CSO.

The assigned KNBS officer accompanied the research team during the mapping process. This involved registering their presence at the chief's or sub-chief's office and briefing them about the study. The chief allocated the research team a village elder and a community health volunteer who were well-known in the community and had previously participated in KNBS activities. Together, they identified selected clusters using KNBS maps of the area and marked the randomly sampled households. Sensitisation of household occupants was carried out during the mapping phase to inform them about the study and its purpose.

Figure S1: Diagram illustrating participants involvement in the studies of schoolchildren and community adults.

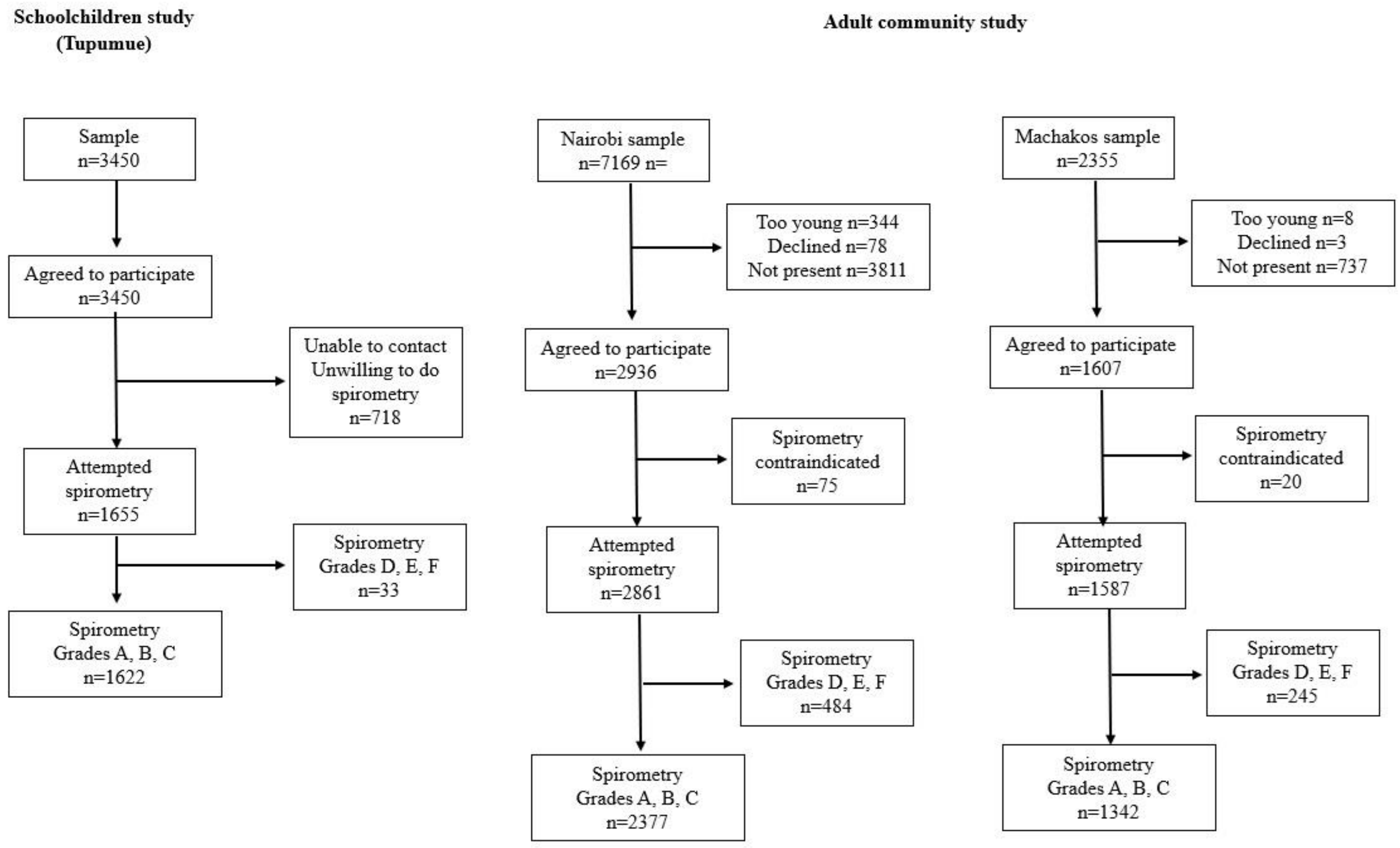

Table S1: Characteristics of those with and without acceptable/reproducible spirometry individual studies

	Nairobi children		Nairobi adults		Machakos adults	
Spirometry	Yes (n=1622) (68.4%)	No (n=751) (31.6%)	Yes (n=2377) (81.0%)	No (n=559) (19.0%)	Yes (n=1342) (83.5%)	No (n=265) (16.5%)
Female n (%)	845 (52.1%)	395 (52.6%)	1491 (62.7%	329 (58.9%)	878 (65.4%)	170 (64.2%)
	p=0.820		p=0.090		p=0.691	
Age, mean (95% CI)	10.8 (10.7,11.0)	10.1 (9.85,10.3)	34.6 (34.1,35.1)	35.5 (34.3,36.7)	47.1 (46.2,48.0)	47.6 (45.5,49.7)
	<0.001		0.157		0.638	
Asset score, median (IQR)	3 (3-5)	3 (3-6)	4 (2-5)	4 (2-5.25)	2 (1-3)	2 (1-3)
	p=0.054		p=0.309		p=0.066	
Wheeze in last 12 months, n (%)	127 (7.9%)	63 (8.4%)	315 (13.3%)	61 (10.9%)	135 (10.1%)	21 (7.9%)
	p=0.657		p=0.136		p=0.283	
Too breathless to dress, n (%)			2 (0.1%)	1 (0.2%)	4 (0.3%)	0
Doctor diagnosed asthma, n (%)	26 (1.6%)	20 (2.7%)	138 (5.8%)	22 (3.9%)	66 (4.9%)	14 (5.3%)0
	p=0.081		p=0.080		p=0.803	

Table S2: FEV_1 and FVC z-scores (GLI-Global) for males and females for the cross-sectional studies

Location (participants)	Nairobi Schoolchildren (n=1622		Nairobi adults (n=2377)		Machakos adults (n=1342)	
	Male (n=777)	Female (n=845)	Male (n=886)	Female (n=1491)	Male (n=464)	Female (n=878)
FEV_1 z-score mean (95% CI)	-0.590 (-0.658, -0.526)	-0.598 (-0.659, -0.538)	-0.405[a] (-0.483, -0.328)	-0.539[a] (-0.591, -0.487)	-0.714 (-0.812, -0.615)	-0.639 (-0.705, -0.574)
FVC z-score mean (95% CI)	-0.682 (-0.745, -0.618)	-0.605 (-0.664, -0.546)	-0.545 (-0.618, -0.472)	-0.550 (-0.600, -0.501)	-0.725 (-0.818, -0.633)	-0.667 (-0.730, -0.604)

CI: confidence intervals

[a]$p=0.004$, all other differences $p>0.05$

Table S3: Characteristics of those with and without acceptable/reproducible spirometry combined community adult studies

	Acceptable spirometry (n=3719)	No acceptable spirometry (n=824)	p value
Machakos, n (%)	1342 (36.1%)	265 (32.2%)	0.033
Female, n (%)	2369 (63.7%)	499 (60.6%)	0.091
Age, median (IQR)	37.0 (26, 49)	36.0 (26, 50)	0.786
Wealth/asset score, median (IQR)	3 (2, 4)	3 (2, 4)	0.467
Wheeze in last 12 months, n (%)	450 (12.1%)	82 (10.0%)	0.083
Dyspnoea hurrying, slight incline, n (%)	300 (8.1%)	62 (7.5%	0.603
Doctor diagnosis of asthma, n (%)	204 (5.5%)	36 (4.4%)	0.195

IQR: interquartile range

Figure S2: Regression lines (95% CI) for GLI-2012 FEV_1 z-scores in children and adult studies in Nairobi and Machakos

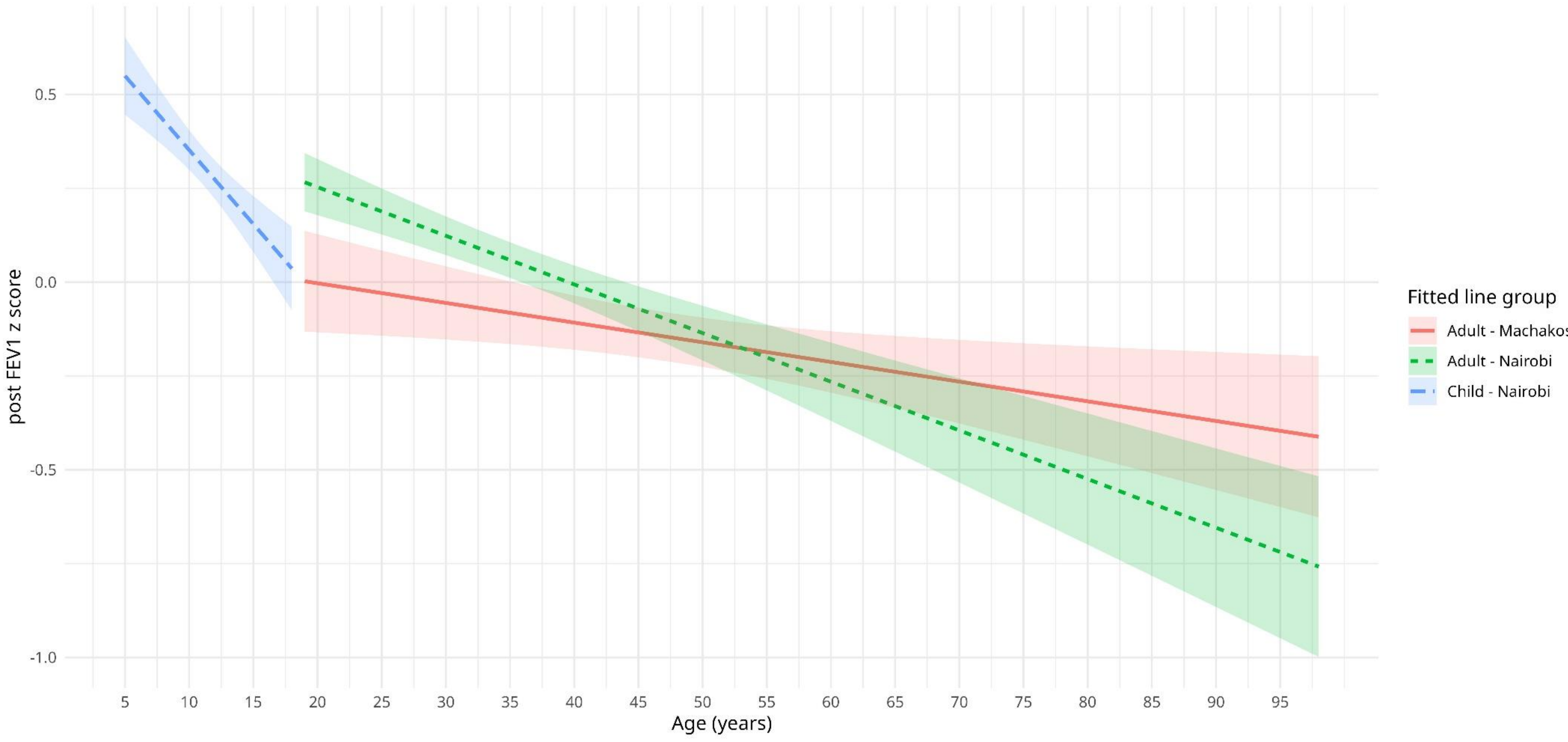

Figure S3: Regression lines (95% CI) for GLI-2012 FVC z-scores in children and adult studies in Nairobi and Machakos

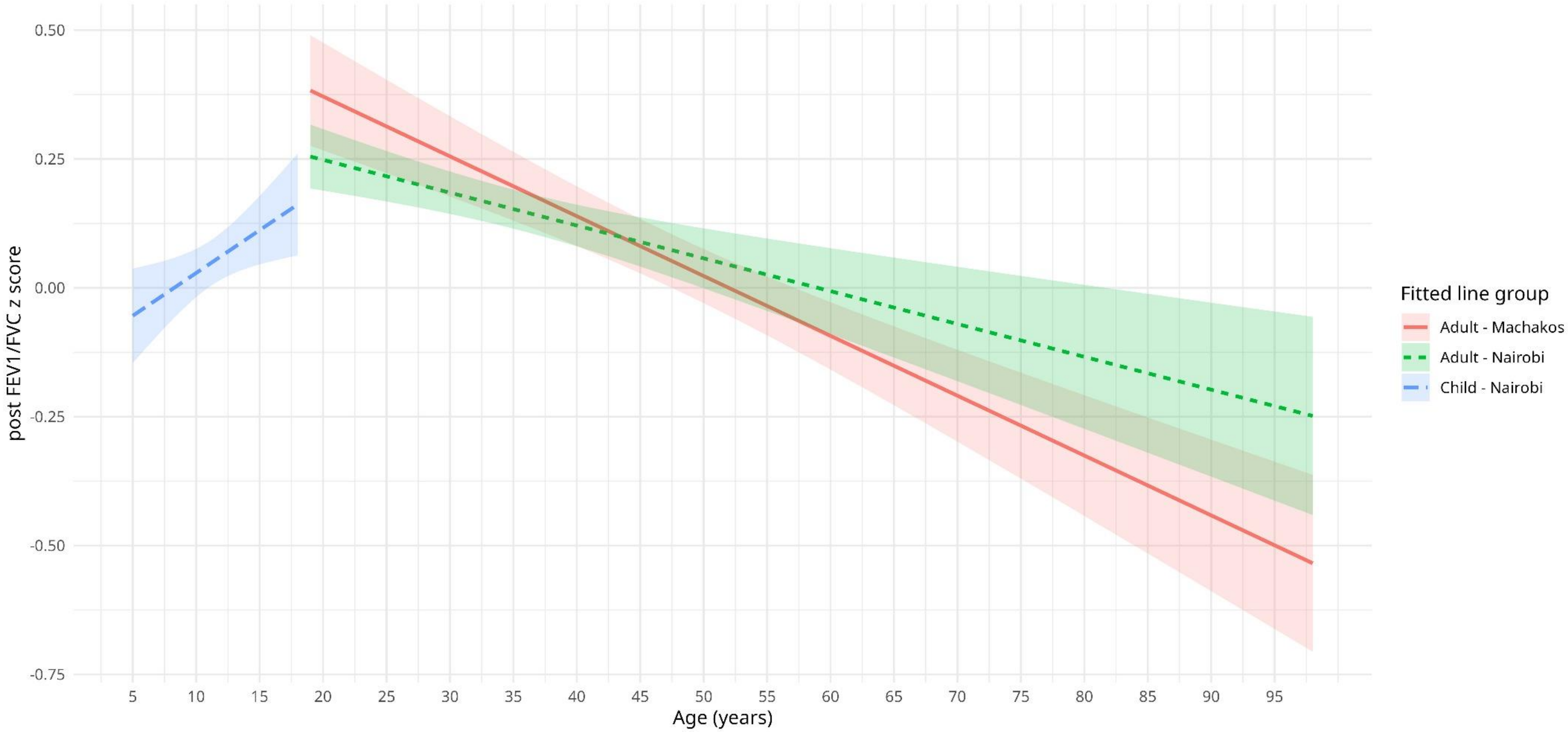

Supplement: Supplementary file 1 [file 01577-2025.SUPPLEMENT.pdf]
